# Research on the Main Properties of Cementitious Mortars Prepared with High-Fe_2_O_3_-Content Raw Drinking Water Treatment Sludge

**DOI:** 10.3390/ma18040759

**Published:** 2025-02-08

**Authors:** Giedrius Girskas, Modestas Kligys

**Affiliations:** Faculty of Civil Engineering, Vilnius Gediminas Technical University (VILNIUS TECH), 10223 Vilnius, Lithuania; modestas.kligys@vilniustech.lt

**Keywords:** drinking water treatment sludge, water sludge, cement mortar, cementitious systems, water absorption of cement concrete, porosity, durability

## Abstract

Drinking water treatment sludge (DWTS) is a typical by-product of drinking water treatment plants. Concerns are growing about how to deal with big amounts of this sludge generated globally. One of the ways is to reuse DWTS as a supplementary material in cementitious systems and thus reduce landfill disposals. For our studies, we used DWTS containing more than 52% Fe_2_O_3_. The DWTS was taken from a water treatment plant in Vilnius, Lithuania. This work aimed to find potential applications of unprocessed DWTS in cementitious systems as a supplementary material that changes the physical and mechanical properties of the final product. Tests were performed with cementitious mortars where the binder was replaced with DWTS (from 0% to 12.5%). Local raw materials such as Portland cement CEM I 42.5R and sand 0/4 were used in the tests. Water absorption, absorption kinetics, and mechanical strength tests were conducted, and predictive freeze–thaw resistance was estimated. The heat release rate and open–closed porosity were also measured. The results showed that DWTS impacts the hydration of cementitious mortars, lowers their density (from 2122 kg/m^3^ to 1954 kg/m^3^), as well as compressive strength (from 41.78 MPa to 24.76 MPa) and flexural strength (from 6.24 MPa to 4.07 MPa), and increases total porosity (from 28.1% to 34.6%) and closed porosity (from 9.1% to 14.9%). The lowest toughness value of 6.08 was recorded in the 12.5% DWTS sample. From our conducted research, it could be observed that raw DWTS potentially changed the porosity parameters of cementitious mortars. This resulted in an incremental improvement in durability and an improvement in the hardness of cementitious mortars. A higher content of raw DWTS changed the colour (to reddish) of cementitious mortars, due to its higher Fe_2_O_3_ content (up to 53%). All of the above-mentioned properties allowed the designing of cementitious landscape products with a wider range of colours.

## 1. Introduction

The intensive use of natural resources and the amount of waste generated are global issues. The transition to a circular economy can be a solution to many of these. The circular economy aims to minimise waste and resources used through the design of advanced products (building materials), product reuse, recycling, and sustainable consumption [1].

With increasing urbanisation and industrialisation, the demand for concrete and mortar keeps growing. The cement industry is resource intensive and big amounts of natural resources are used to produce concrete mixes and products. There are many supplementary materials (ground glass, waste rubber, crushed waste concrete, waste marble, concrete construction and demolition waste, etc.) that can be used as aggregates in cementitious systems to minimise the depletion of natural resources [2,3,4,5]. Various pozzolanic additives (natural or synthetic), fly ash, bottom ash, etc., are available as binder supplements [6,7].

DWTS is generated after the treatment of potable water supplied to urban residents. Expanding cities, growing food industries, and rising living standards increase the demand for drinking water [8]. DWTS waste is generated in relatively large quantities. The conventional potable water purification process involves coagulation, flocculation, sedimentation, filtration, and disinfection. This process generates a large amount of drinking water treatment sludge (DWTS) [9].

The chemical composition of this sludge varies by country. There are several other aspects to be considered. First, the source of water, i.e., whether it is raw water (from rivers, lakes, reservoirs, or mountains) or from surface boreholes (wells) or deep boreholes. Second, the chemical substances used for water filtering should be evaluated (i.e., AlCl_3_, FeCl_3_, FeCl_2_, and FeSO_4_⋅7H_2_O). Some types of DWTS have high concentrations of Al_2_O_3_ and SiO_2_, which create pozzolanic activity in cementitious systems [10,11,12]. Other types of sludge have higher concentrations of iron or other minerals.

DWTS is widely used in the ceramic industry, mainly as a pigment [13,14,15]. In addition to pigmentation, DWTS improves the compressive strength properties of ceramic items up to 10% [16]. Results obtained by Benlalla and co-authors show that the compressive strength of ceramic samples increases up to 30% when fired at 1000 °C [17].

Gomes and co-authors replaced fine aggregate with DWTS and found that up to 5% of the fine aggregate can be replaced (the incorporation of 5 percent of wet sludge reduces approximately 50 percent of the concrete compression strength). They also noted the change in colour of the final products, which became brownish/pinkish [18].

Tafarel and co-authors tested concrete in which fine aggregate was replaced with water treatment sludge. They found that, at 28 days, the mechanical strength of concretes containing 5% water treatment sludge by weight of cement was 11% higher. Water absorption tests showed a 12% higher absorption rate in the samples containing 5% sludge and 32% higher water absorption in the samples modified with 10% water treatment sludge [19].

DWTS has a direct effect on cement hydration processes in cementitious systems as it reduces the heat released and delays hydration reactions [20]. Ruviano and co-authors suggest that DWTS containing small amounts of Al slows down the development of hydration products, whereas Al-rich DWTS can accelerate hydration reactions [21]. DWTS also influences the initial and final setting times of cement paste. Ng and co-authors tested the initial and final setting times and found that DWTS added at 15% increased the setting time from 6.3 to 36 h, thus confirming the findings of other authors that DWTS inhibits cement hydration [22].

The durability of construction materials depends on their porosity and absorption. In capillary absorption tests, Duan and co-authors observed the lowest capillary absorption in samples modified with 20% DWTS [23]. Ng and co-authors achieved similar results and reported lower capillary absorption and a better pore structure in the mortars where cement was replaced with DWTS of to 6%. Other researchers studied self-compacting concrete mixes and found that DWTS can be added at up to 15% to produce self-compacting concrete and that DWTS can be used to reduce the depth of water penetration [24].

Our study was focused on the usage of an Fe_2_O_3_-rich (content up to 53%) raw DWTS in cementitious mortars. A review of literature sources showed that raw DWTS contains large amounts of SiO_2_ and Al_2_O_3_, which could give a pozzolanic effect for cementitious mortars. The raw DWTS which was studied in our research had relatively small contents of SiO_2_ and Al_2_O_3_, but contained more than 53% Fe_2_O_3_. It should be noted that it was a local natural water sludge, obtained in the water treatment plants without any chemical treatment, which was radically different from other water sludges analysed by other authors. A review of literature sources also analysed the influence of the introduction of DWTS on the changes in colour range of cementitious mortars. A large content of DWTS had a direct effect on the colour change of cementitious mortars, and this fact allows us to expand the possibilities of the use of raw DWTS. The aim of this work was to determine how larger amounts of raw DWTS could influence the key (physical, mechanical, durability) characteristics of cementitious mortars.

## 2. Materials and Methods

### 2.1. Materials

Sand of the following characteristics—fraction 0/4, particle density of 2650 kg/m^3^, fineness modulus 4.0, type of fines f_3_—was used as fine aggregate. The mineral composition of sand was as follows: 62.8% quartz; 18% carbonates; 15.6% feldspars; 3.7% other.

Portland cement CEM I 42.5R was used as a binder. Calcium and silicon oxides are the prevailing compounds in cement. They account for more than 80% of the total oxide content: CaO—61.4%; SiO_2_—19.5%; Al_2_O_3_—5.0%; Fe_2_O_3_—3.1%; MgO—3.9%; SO_3_—2.5%; K_2_O—1.1%; N_2_O—0.1%.

CEM I 42.5R has the following mineral composition: C_3_S—57.8%, C_2_S—22.15%, C_3_A—6.65%, C_4_AF—13.40%.

**Figure 1:**
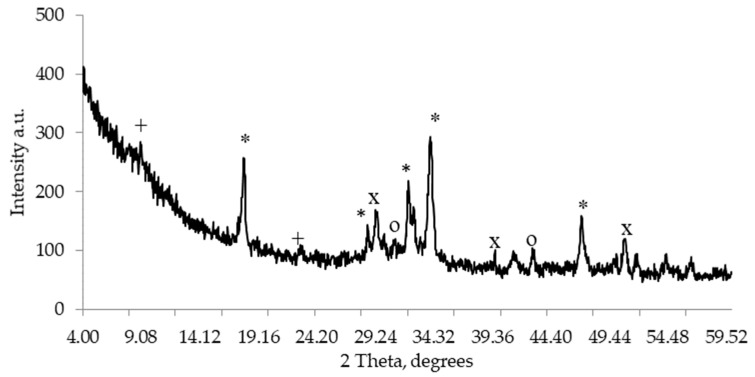
XRD of hardened cement +—ettringite; *—portlandite; X—calcite; O—alite.

**Figure 2:**
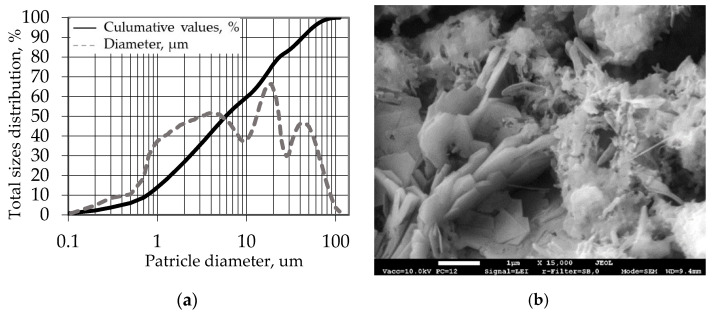
Properties of Portland cement CEM I 42.5R: (**a**) particle size distribution; (**b**) SEM magnification × 15,000.

An image of hardened cement paste XRD is presented in Figure 1. Ettringite, portlandite, alite, and calcite were detected in the paste. Figure 2a illustrates the particle size distribution of CEM I 42.5R. The binder has an average particle size of 14.24 µm and specific density of 3150 kg/m^3^. An SEM image of hardened cement paste is presented in Figure 2b. The cement matrix is dense, Ca(OH)_2_ crystals are evenly distributed and fill the gaps of C-S-H, and plenty of ettringite minerals fill the pores of the cement matrix.

The drinking water treatment sludge was taken from a water treatment plant in Vilnius, Lithuania. The sludge was taken in the wet state. It was initially dried in a 90 ± 5 °C fan dryer until a constant mass was reached, i.e., all the water evaporated.

The chemical composition of the DWTS was as follows: Fe_2_O_3_—52.6%; CO_2_—28.1%; P_2_O_5_—6.8%; CaO—6.22%; SiO_2_—4.74. Apparently, Fe_2_O_3_ prevails. It should be noted that the DWTS used in our tests contained a small amount of SiO_2_ at 4.74% and a very small amount of Al_2_O_3_ at 0.127%.

**Figure 3:**
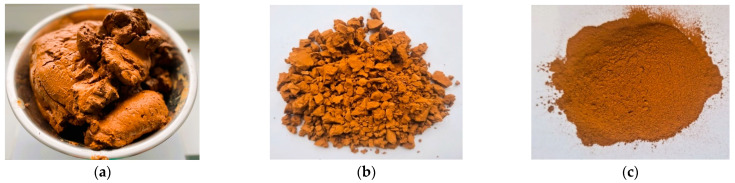
Drinking water treatment sludge (DWTS): (**a**) raw; (**b**) dried; (**c**) dried/milled.

The image in Figure 3a is of water-saturated DWTS taken from the water treatment plant. An image of the DWTS dried to the constant mass is presented in Figure 3b. The dried sludge was lumpy; therefore, the lumps had to be crushed to the fineness of cement particles. The image in Figure 3c is of sludge crushed in the ball mill.

The DWTS was ground/milled in a drum mill with ceramic milling bowls using a dry process. The milling lasted for 48 h.

### 2.2. Cement Mortar Compositions

Cement mixes were designed to investigate the effect of DWTS on cementitious systems. DWTS was added in increments up to 12.5% by weight of cement. Compositions of cement mixes modified with DWTS are presented in Table 1.

Concrete mixtures were mixed in a forced action mixer compliant with EN 196-1:2016 requirements [24]. The total mixing time did not exceed 3 min. The samples were formed in watertight and non-absorbent moulds of dimensions 40 × 40 × 160 mm. The paste was poured in two layers and compacted on a vibrating table. The samples were covered with a waterproof film and kept in the moulds at an ambient temperature of 20 ± 2 °C for 24 h. After 24 h, the samples were demoulded and cured in water of the same temperature. The curing time was 28 days.

Six formulations of mortars were mixed: a control composition and 5 compositions with different DWTS contents where up to 12.5% cement was replaced with DWTS. W/C rate was kept constant at 0.55. Compositions of the mixes are presented in Table 1.

### 2.3. Test Methods

The temperature of exothermic reactions during the hydration and curing of cement mortars was measured according to the Alcoa Industrial Chemicals test method [25]. The mixed cement mortars were immediately placed in watertight and moisture-proof moulds made of polyethylene terephthalate in the shape of a truncated cone with a capacity of 200 mL. The mortars were compacted for 10 s on a vibrating table. The sample weight was 400 g. The filled mould was placed into an insulation box with a thermocouple inserted in the centre of the sample. Temperature fluctuations in the mix over time were registered using a data logger PICO TC-08 (Pico Technology, Cambridgeshire, UK) connected to the thermocouple and a PC (DELL Inspiron 3520 15 i5-1235U/16/1TB SSD/W11, Dell Technologies, Round Rock, TX, USA). The test lasted for 30 h (1800 min).

**Figure 4:**
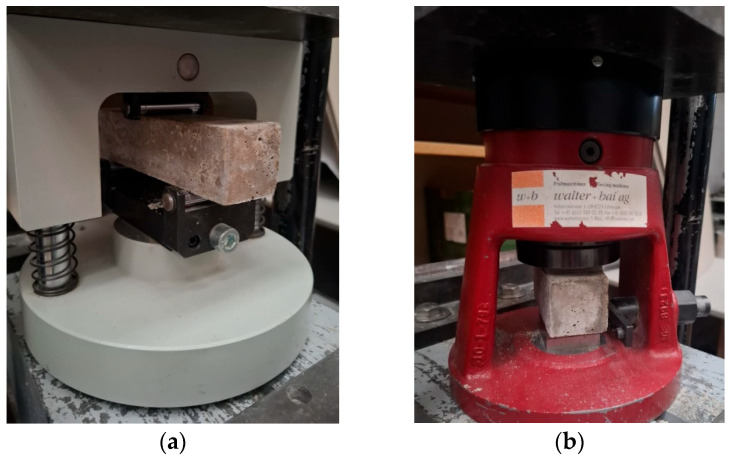
Mechanical tests of the mortar: (**a**) flexural strength of mortars; (**b**) compressive strength of mortars.

The flexural strength of hardened mortar was tested according to EN 12390-5:2019 [26]. A 3-point system was used for the bending test with a loading rate of 0.04 kN/s. The flexural strength of 4 prism-shaped samples of each mortar mixture was tested (Figure 4a).

The flexural strength was calculated by the following equation:

(1) $$f_{f}=1.5\frac{F\cdot l}{b\cdot d²}$$

where *f_f_* is flexural strength, MPa; *F* is the maximum flexural force, N; *l* is the distance between the supports, mm; *b* is the width of the mortar sample, mm; *d* is the thickness of the mortar sample, mm.

The compressive strength was tested according to EN 12390-3:2019 at the loading rate of 1.0 kN/s using a hydraulic press (Figure 4b) [27].

The compressive strength of 8 samples of each mortar mixture was tested using the prism halves remaining after the flexural strength tests. The compressive strength was calculated by the equation:

(2) $$f_{c}=\frac{F}{A}$$

where *f_c_* is compressive strength, MPa; *F* is the maximum compressive force, N; *A* is the cross-sectional area of the sample, mm^2^.

The determination of water absorption was carried out using the methodology given in the EN 13369:2018 standard [28]. A total of 18 samples were used for this study, i.e., 3 from each batch. The water absorption of the mortar was calculated using the formula:

(3) $$W=\frac{m1-m2}{m2}\cdot 100\%$$

where *W*—water absorption of the samples%; *m*_1_—mass of the soaked sample, g; *m*_2_—mass of the dried sample, g;

The open porosity of the mortar was measured using a modified EN 1015-10:2002 method. Wet samples were weighed after the scheduled curing period. Excess water was wiped with a damp cloth from the surface and the samples were weighted in water with air bubbles removed from the sides. Afterwards, the weighted specimens were dried in a drying oven at a temperature of 100 ± 5 °C until constant mass was achieved [29].

Total porosity was determined from the equation:

(4) $$P_{t}=(1-(\;\frac{T}{2690}\;))\;\times \;100,\;\%$$

where *P_t_* is total porosity, %; *T* is bulk density (kg∙m^3^).

Open porosity was determined from the equation:

*P_o_* = *W*_*p(t)*_, % (5)

where *P_O_* open porosity, %; *Wp_(t)_* is water absorption by volume, %.

Closed porosity was determined from the equation:

*P_c_* = *P_t_* − *P_o_*, % (6)

Frost resistance of concrete depends both on open porosity (the amount of capillary pores) and on closed porosity (air content in the mixture), and can be determined quantitatively by the frost resistance coefficient *K_F_*, which is derived from the equation [30]:

(7) $$K_{F}=\frac{P_{u}}{0.09\;P_{a}}$$

where *P_u_* is the closed porosity of concrete; *P_a_* is the open porosity of concrete.

Knowing the value of the frost resistance coefficient *K_F_*, the freeze–thaw resistance of hardened mortar can be predicted according to the function of the mortar’s freeze–thaw resistance and the frost resistance coefficient *K_F_*.

The toughness of hardened mortar samples was calculated at 7, 28, and 90 days using the results of the flexural and compressive strengths of the mortar samples from the equation [31]:

(8) $$k=\frac{f_{c}}{f_{f}}$$

where *k* is toughness; *f_c_* is the compressive strength, MPa; *f_f_* is the flexural strength, MPa.

**Figure 5:**
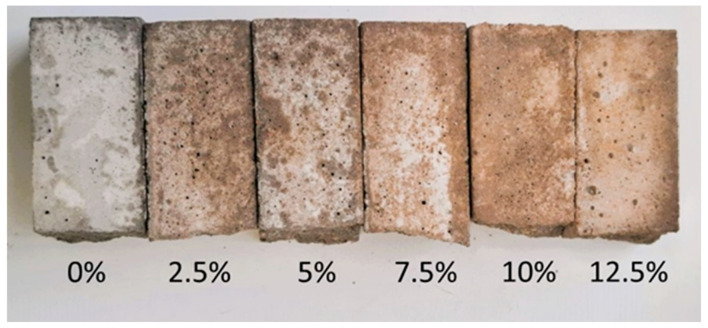
Cement mortar samples with different amounts of DWTS.

Images of 40 × 40 × 160 mm beam halves after flexural tests are presented in Figure 5. The colour of mortar samples changes with the increasing content of DWTS added up to 12.5%. The samples become reddish due to the red/orange colour of DWTS, as seen in Figure 5.

## 3. Results and Discussion

### 3.1. Properties of Hydration

Portland cement hydration is a process of chemical reactions between clinker minerals, calcium sulphate, and water [32]. As a result of these reactions, the cement paste binds, hardens, and acquires mechanical properties [32,33]. Cement hydration involves five well-defined phases [34]. We analysed four phases because Phase 5 is a steady slow-down phase which follows the hydration reactions.

**Figure 6:**
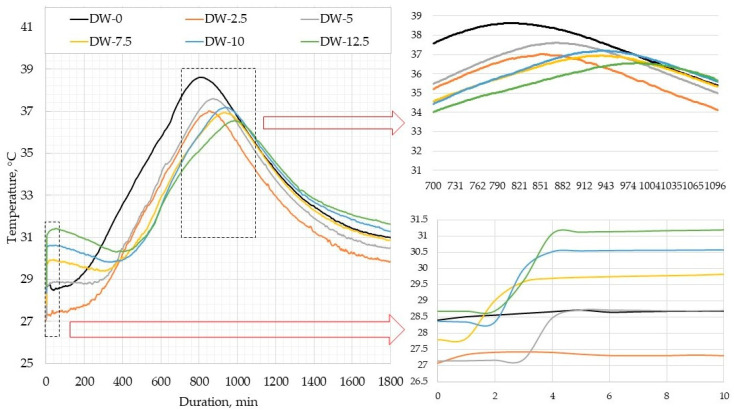
Heat release in DWTS-modified cement mortars.

Figure 6 illustrates the curves of heat release in DWTS-modified cement mortars. In Phase 1, when cement is mixed with water, cement particles become wet, crystals of cement minerals start melting, and a small amount of heat is released. In our tests, this phase lasted for 4–5 min. No temperature peaks were observed in the control composition sample and the sample containing 2.5% DWTS. However, with a higher additive content the temperature rose from 1.5 °C to 3 °C in the first min. Later, during the induction period, this process slowed down. A high concentration of ions was achieved, cement minerals continued melting, and the formation of hydrocarbon crystalline structures began. The duration of Phase 2 in our tests was different. It lasted 150 min in the control sample. In the sample containing 2.5% DWTS, it extended to 210 min. The increase of DWTS content up to 5% extended the duration of Phase 2 to 270 min. Higher amounts of the additive extended the induction period even more: 330 min in the sample with 7.5% DWTS and 420 min in the samples with 10% and 12.5% DWTS.

The induction phase (Phase 2) is followed by a new significant heat liberation phase, during which cement hydrates become crystallized. These processes were observed in our research (Figure 6). This phase marks the formation of different cement minerals and early strength development. As mentioned before, the induction period extended with a higher content of DWTS. The peak temperature in the samples of all compositions was reached in 600–630 min, i.e., the period of temperature rise. However, the peak temperature values varied. The highest peak temperature 38.6 °C was recorded in the sample of control composition. The sample containing 2.5% DWTS released heat of 37 °C. The sample containing 5% DWTS had a slightly higher temperature of 37.6 °C. The peak temperatures of the samples modified with 7.5% and 10% DWTS were 37.1 °C and 36.9 °C, respectively. The lowest heat release temperature of 36.5 °C was recorded in the sample modified with 12.5% DWTS. The explanation for the decrease in the peak temperatures with the increase of DWTS content in mortar formulations is that the sludge additive has no effect on the hydration of cementitious systems and thus does influence the temperature rise. As the induction period (Phase 2) extended with a higher DWTS content, the peak temperatures were also achieved later.

The increased content of DWTS results in the delay and the decrease of the peak temperatures due to the complex effect of various substances in it. Firstly, as described by the water film fluid (How) model, organic matter can change the Hamaker constant of the water film and reduce the transfer rate of water. When the Hamaker constant is positive, the conjoining pressure makes the film unstable and inhibits the transfer of water to supply the need of cement hydration (this also leads to the reduced heat release) [35].

Secondly, the presence of Fe_2_O_3_ slows down the main hydration reactions of Portland cement (with extension of the induction period and delaying of the peak of hydration temperature) due to the incorporation of Fe ions into the hydration products (ettringite) of Portland cement. Higher Fe dosages may suppress ettringite formation due to the instability of Fe ions in the crystal structure. This effect is more pronounced when Fe_2_O_3_ is combined with other oxides [36,37].

Thirdly, phosphoric acid and phosphates can form precipitates on the surface of hydrating cement particles, reducing the dissolution rate of cement phases and affecting the nucleation process of hydrates [38].

Opinions regarding the use of DWTS in cementitious systems vary. Some authors argue that DWTS has a negative effect on the early hydration of cement mixes [20,39]. Like Gomes and co-authors, we also saw in our tests that with a higher DWTS content in modified mortar samples the temperature of heat released became lower and the peak temperatures were reached with delay. According to Gomes et al., when DWTS is added at 5–10%, the delay of peak temperature is 100–280% [34]. Similar trends were also observed by other authors [39,40].

The period of accelerated hydration is followed by a prolonged period of slow reactions. In our case, this phase lasted for 30 h (1800 min). It should be noted that the lowest temperature throughout the test was recorded in cementitious mortars containing 2.5% DWTS. In the last phase when the temperature drops, we observed that the temperature drop was slower in the mortars with a higher DWTS content. This trend is illustrated in Figure 6. After 1800 min of testing, the temperature of the mortars containing 12.5% DWTS was 31.6 °C compared to 29.8 °C in the mortars modified with 2.5% DWTS.

The generally accepted explanation for the test results above is that organic matter in DWTS directly affects the hydration process of cement paste. Hydrophobic organic matter delays the dissolution of cement clinker in the initial reaction phase, and alkali ions in DWTS may also inhibit the normal hydration of calcium silicate. We can say that DWTS lacks pozzolanic reactivity whereas the organic matter in DWTS usually affects cement hydration due to its hydrophobicity, resulting in poor development of mechanical properties.

### 3.2. Density

**Figure 7:**
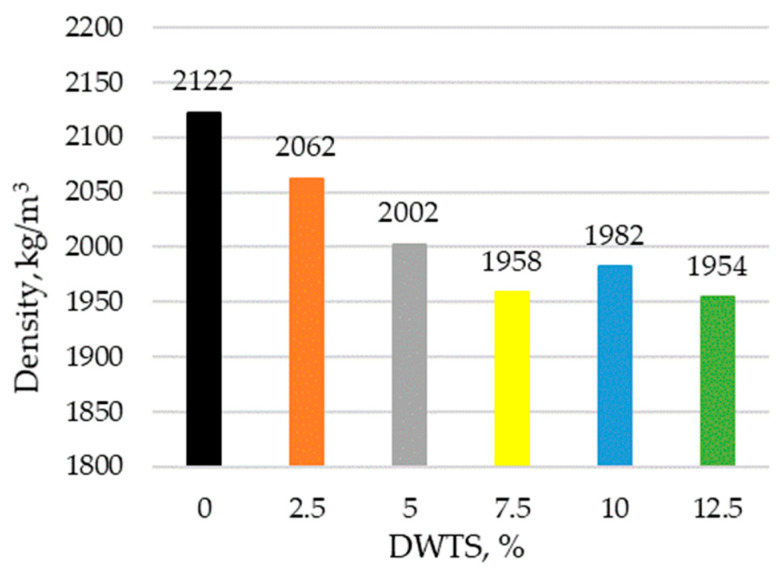
Density of mortar with DWTS.

The addition of DWTS into cementitious mortars reduced their density (Figure 7). The density of the control sample was 2122 kg/m^3^. With the addition of DWTS at 2.5% by weight of cement, the density of the modified mortar decreased to 2062 kg/m^3^ or 3%. With a higher DWTS content of 5% and 7.5%, the density continued decreasing to 2002 kg/m^3^ and 1958 kg/m^3^, respectively.

Modified mortars with the highest DWTS content of 12.5% had a similar density of 1954 kg/m^3^ along with the mortars containing 7.5% DWTS. Thus, when 12.5% cement is replaced with DWTS, the density reduces by 7.9%.

### 3.3. Water Absorption

**Figure 8:**
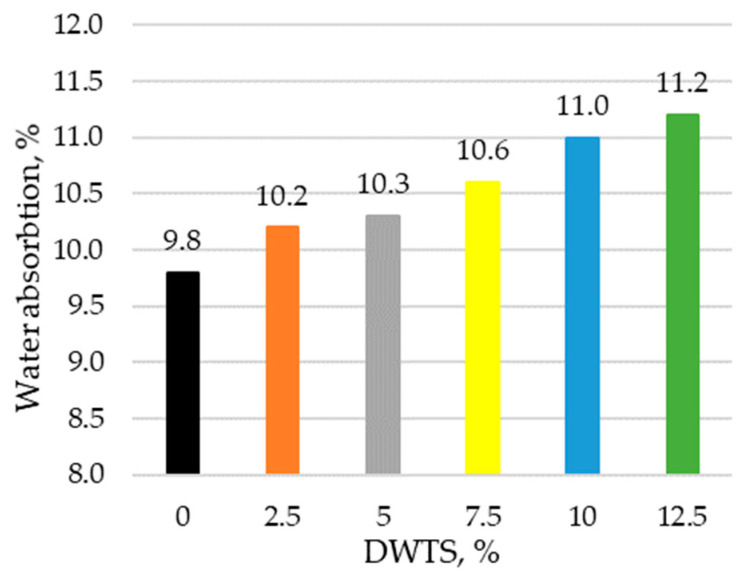
Water absorption of mortar with DWTS.

Water absorption is mainly obtained by capillary water absorption and immersion tests. Water absorption test results are presented in Figure 8.

The results show that water absorption increases in modified mortars with a higher DWTS content. With the addition of DWTS at 2.5%, water absorption increases by 4%. Compared to 2.5% DWTS, there is a slight increase in water absorption in modified mortar samples containing 5% DWTS, with water absorption values ranging from 10.2% to 10.3%, respectively. The water absorption rate increased in proportion to DWTS content in the mortar and reached 11.2% in the samples where cement was replaced with 12.5% DWTS.

The increase equalled 14.2% compared to the control sample. Density correlates with water absorption. If density decreases, water absorption increases accordingly because more pores are formed.

### 3.4. Kinetics of Water Absorption

**Figure 9:**
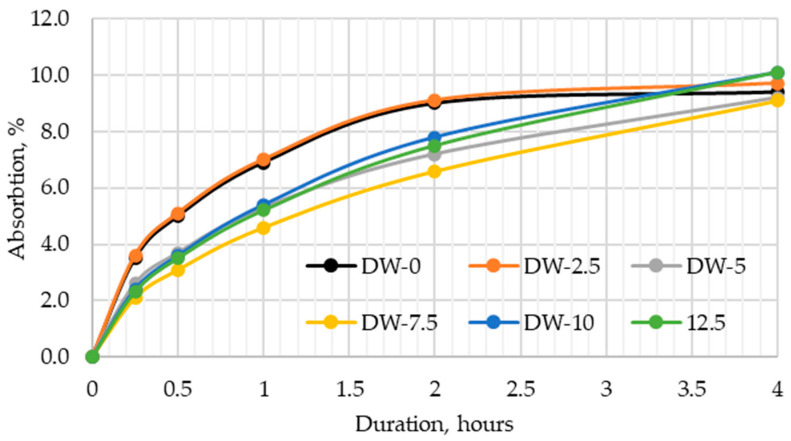
Water absorption kinetics of modified mortar with DWTS.

The tests on water absorption kinetics in DWTS-modified cement mortars (Figure 9) showed that in the first 4 h, the absorption of water varied between samples. The control sample and the sample modified with 2.5% DWTS showed different water uptake in the first 2 h compared to the samples of other compositions. Both the control and the 2.5% DWTS sample had a faster water absorption rate in the first 2 h, with water uptake observed from the first 15 min until the second h. At the third and fourth h, the water absorption rate became similar to other samples. The results in Figure 8 show that the control sample had the lowest water uptake. A trend of a higher water absorption rate and faster kinetics with a higher DWTS content was observed. This is due to the fact that raw DWTS is introduced, which negatively affects the properties.

Raw DWTS contains organic impurities that negatively affect the hydration products of cement, decreasing density and increasing absorption.

### 3.5. Porosity

**Figure 10:**
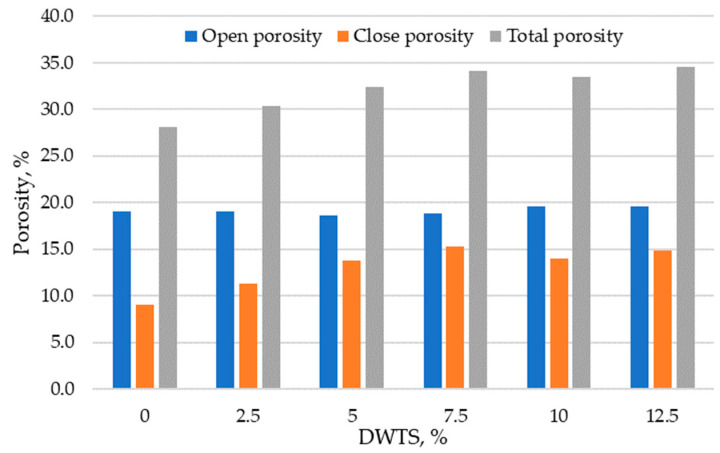
Porosity of modified mortar with DWTS.

Porosity and pore size distribution are the key characteristics that can be used to determine the durability of modified cementitious systems. Figure 10 presents the results of total, open, and closed porosity tests in cementitious mortars modified with DWTS of up to 12.5%. The results show that the control sample had an open porosity of 9%, total porosity of 28.1%, and closed porosity of 9.1%. The samples modified with 2.5% DWTS had the same open porosity of 9%, but the closed porosity increased to 12.4%. Modified mortar samples containing 5% DWTS showed that closed porosity increased to 13.8% and was 51.6% higher compared to the control composition. The open porosity reduced from 19% to 18.6%. The highest closed porosity value of 15.3% was recorded in the samples modified with 7.5% DWTS compared to only 9.1% in the control samples. The total porosity of 7.5% DWTS samples was 18.8%. With the increase of DWTS content from 10% to 12.5% the open porosity increased to 19.6% and the closed porosity was 14% and 14.9%, respectively.

Ng and co-authors investigated water absorption in cementitious systems where only a small amount of cement (<6%) was replaced with CWTS. They reported that CWTS optimises the pore structure of the mortar and reduces water absorption [22]. Kaish and co-authors investigated self-compacting concrete. They found that 15% CWTS added by weight of cement reduced the depth of water penetration [41]. Water absorption in concretes increases with increasing open porosity. Owaid tested concrete containing 15% of water treatment sludge after 28 days of curing. The tested samples showed 28.5% lower water absorption compared to the control composition [42]. According to Ahmad, CWTS added to cementitious mortars increases the porosity of the mortars and subsequently increases water absorption [31].

### 3.6. Mechanical Properties

**Figure 11:**
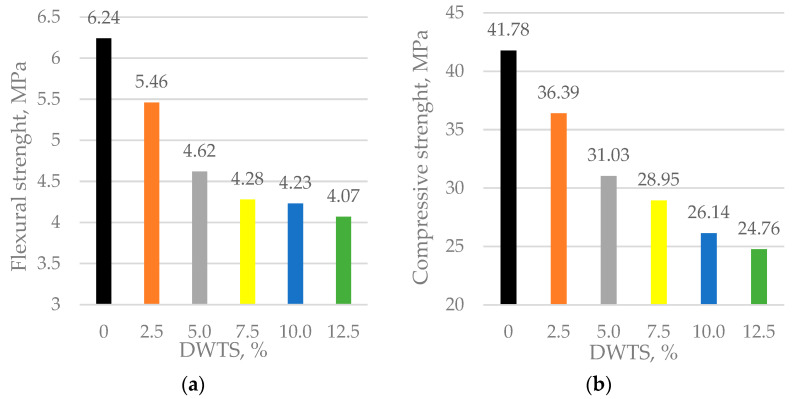
Mechanical properties of modified mortar with DWTS: (**a**) flexural strength; (**b**) compressive strength.

Tests on mechanical properties (Figure 11a,b) showed a tendency for both flexural and compressive strengths to decrease with increasing DWTS content. The decrease in flexural and compressive strengths of cementitious mortars firstly depends on the rapid decrease of their density (see Figure 7). As was pointed out by [43], organic matter helps to stabilize air bubbles, entrained during the mixing process of cementitious mortars.

Calcium silicate hydrate (C-S-H) is a mesoporous amorphous primary binding phase (with water confined in the gel pores), which controls the mechanical and chemical properties of cementitious materials and can be influenced by the organic matter. The reduction in the stiffness and cohesive force of the C-S-H gel may occur due to the breakage of silicate chains and the penetration of water molecules with the presence of the organic matter in the cement paste [44]. The organic matter also influences the strength development of cementitious mortars largely by inhibiting the formation of pozzolanic reaction products (such as the same C-S-H). The degree of inhibition depends on the type of pozzolanic reaction; thus, the formation of C-S-H-2 was less inhibited by organic matter than was the formation of C-S-H-1 [45].

We found that, at 28 days, the flexural strength of the control sample was 6.24 MPa. With only a small amount of DWTS added at 2.5%, the flexural strength drops by about 12.5% to the value of 5.46 MPa. In 5% and 10% DWTS samples, the flexural strength reduced to 4.62 and 4.23 MPa, respectively. When DWTS content in the mortar mix is increased to 12.5%, the flexural strength drops to 4.07 MPa and is 34.7% lower than the strength of the control sample.

The same trends in decreased values were observed in compressive strength. The control sample had a compressive strength of 41.78 MPa. A significant decrease of 12.9% to a compressive strength value of 36.39 MPa was observed in the samples modified with 2.5% DWTS. The 5% DWTS samples had a compressive strength of 31.03 MPa. The trend of decreasing compressive strength was observed with the increase of DWTS content to 12.5%: 28.95 MPa in 7.5% DWTS samples, 26.14 MPa in 10% DWTS samples, and 24.76 MPa in 12.5% DWTS samples. The 12.5% DWTS samples had 40% lower compressive strength than control samples.

The same findings were obtained by other researchers [46]. They argue that DWTS with a high iron content has a negative effect on the mechanical properties of concrete. The compressive strength of concrete at 28 days decreased from 41.0 MPa to 13.5 MPa (about 67%), with the replacement of Portland cement with DWTS from 0% to 2%, respectively. The tendency of the reduction of flexural strength was similar with the results of compressive strength. The flexural strength of concrete at 28 days decreased from 3.0 MPa to 1.5 MPa (about 50%), with the replacement of Portland cement with DWTS from 0% to 2%, respectively. The possible reason is high water absorption and the considerably high organic content of dried DWTS, which hinders the formation of silicate or calcium silicate hydrate (C-S-H). Researchers [47] also argue that dried iron-based DWTS has a high organic content, so the compressive strength of the cement paste decreases with adding dried iron-based DWTS, even though its content is lower than 10% [47]. They stated that after 28 days of hardening the compressive strength of cement paste reduced from 75 MPa to 50 MPa, with the addition of DWTS from 0% to 10%, respectively. Our results of compressive and flexural strength tests are confirmed by the findings of other authors [40]. They found that after 28 days of hardening compressive strength reduced from 27 MPa to 10 MPa, with the addition of DWTS (from 0% to 30%), respectively. The same tendency was fixed with flexural strength results after 28 days of hardening. Flexural strength reduced from 5.0 MPa to 3.5 MPa, with the addition of DWTS (from 0% to 30%), respectively. The decrease in compressive and flexural strengths was 63% and 30%, respectively.

To eliminate organic content and improve the performance of cement-based composites, DWTS was treated at elevated temperatures before it was used in concrete [48,49,50].

### 3.7. Frost Resistance

**Figure 12:**
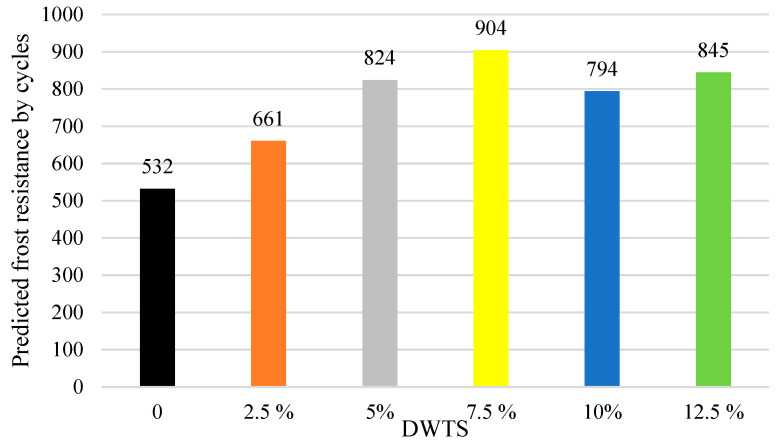
Predicted frost resistance (cycles).

The predicted resistance of concrete to freezing and thawing cycles was determined by using the freeze–thaw durability factor. The relationship between the predicted resistance to freezing and thawing cycles and DWTS content is presented in Figure 12. The data in Figure 12 show that the predicted resistance to freezing and thawing is 532 cycles in the control sample.

The predicted resistance to freezing and thawing increases to 660 cycles in 2.5% DWTS samples, i.e., more than 24%. Such an increase is related to higher closed porosity (Figure 10). The predicted frost resistance increases to 824 cycles in 5% DWTS samples. The maximum number of 904 cycles is reached when 7.5% of cement is replaced with DWTS. In the case of the 7.5% DWTS sample, the same upward trend is observed in Figure 10 showing the increased closed porosity. At a higher DWTS content, the number of freezing and thawing cycles grows from 793 to 844 in 12.5% DWTS sample.

### 3.8. Toughness

**Figure 13:**
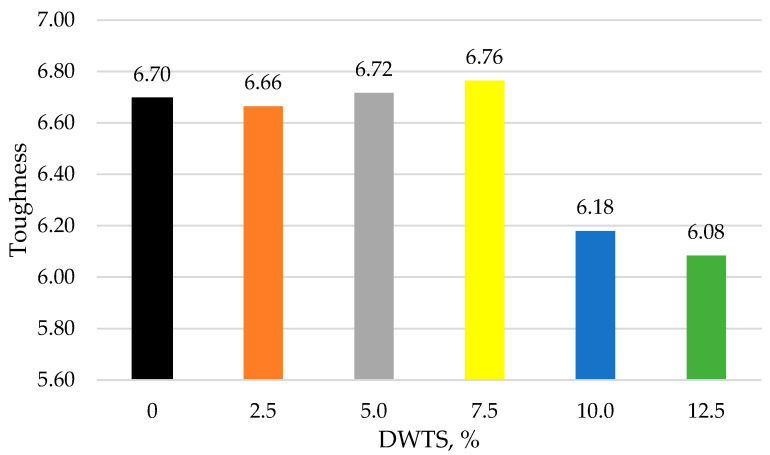
Ratio of compressive strength to flexural strength (toughness) at different amounts of DWTS.

The ability of cementitious mortar to absorb energy and deform without fracturing (crucial for enhancing the durability and performance of the structures) is called toughness. As can be seen from Equation (8), it strongly depends on the ratio of compressive strength to flexural strength. According to [51,52], the lower the ratio, the better is the toughness. Figure 13 shows the ratio of compressive strength to flexural strength at different DWTS contents. The toughness value of the control sample is 6.69. The toughness increases to 6.71 when 7.5% DWTS is added to the mix, whereas the 10% DWTS sample has a toughness of 6.18. In comparison to the control sample, the toughness of 10% DWTS sample was reduced by 7.62%. The lowest toughness value of 6.08 was recorded for the 12.5% DWTS sample.

The sharp decrease in the compressive strength and slight increase in toughness with an increased DWTS content could be explained by the constant W/C ratio (see Table 1), which determined a higher porosity (as well as lower density) as the cement content decreased. This change in porosity has a higher impact in the case of uniaxial loading situations as encountered during the tests of cementitious mortars [VIII], leading to significant changes in compressive strength for increasing DWTS content, when compared with the changes in toughness.

The same constant W/C ratio resulted in a sharp decrease of flexural strength with an increased DWTS content. However, the smaller changes in the flexural strength, when compared with the decrease in the toughness with increased DWTS content (especially when the DWTS content was up to 10%) indicates the improvement in the interface properties of cementitious mortars by the addition of DWTS. These interface properties have a significant effect on the tensile-loading performance of cementitious mortars.

## 4. Conclusions

The effect of Fe_2_O_3_—rich DWTS on the properties of cementitious mortars was investigated. CEM I 42.5 R cement was replaced with up to 12.5% DWTS. The following results were obtained:

DWTS impacts the hydration of cementitious mortars. Heat release tests showed that the induction phase in the control sample lasted for 150 min. In the sample containing 2.5% DWTS, it extended to 210 min. The increase of DWTS content up to 5% extended the duration of induction phase to 270 min. Higher amounts of the additive extended the induction period even more: 330 min in the sample with 7.5% DWTS and 420 min in the samples with 10% and 12.5% DWTS.

1. The incorporation of DWTS in cementitious mortars reduces their density. The density of the control sample was 2122 kg/m^3^. The addition of 2.5% of DWTS by weight of cement caused a 3% decrease in density. The density of 5% DWTS and 7.5% DWTS samples decreased by 5.6% and 9.6%, respectively. The density of 12.5% DWTS samples decreased by 7.9%.
2. The addition of DWTS to cementitious mortars increases the water absorption of the mortar. With the addition of DWTS at 2.5%, water absorption increased by 4%. Water absorption rate increased in proportion to DWTS content in the mortar and reached 11.2% in the samples where cement was replaced with 12.5% DWTS. The increase equalled to 14.2% compared to the control sample. Water absorption kinetics revealed that the control sample and 2.5% DWTS sample behaved differently from the samples of other compositions. The samples of the first two compositions showed a more abrupt water uptake effect.
3. Modified mortar porosity tests showed that the total porosity increased with the increase of DWTS content in cementitious systems. The porosity of the control sample was 28.1% whereas the porosity of the sample with the highest DWTS content was 34.6%. The open porosity ranged from 18.6% to 19.6%. The closed porosity of modified mortars increased with the increase of DWTS content in the mortar mix. The closed porosity of the 2.5% DWTS sample was 51.6% higher compared to the control composition. The highest closed porosity value of 15.3% was recorded in the samples modified with 7.5% DWTS compared to only 9.1% in the control samples. Higher amounts of DWTS lead to the formation of more pores in cementitious systems, resulting in higher water absorption. However, it can be assumed that with higher amounts of closed pores, these mortars will be more resistant to numerous freeze–thaw cycles and thus have better durability characteristics.
4. Estimations of predictive freeze–thaw resistance showed that closed porosity has a positive effect on frost resistance. The maximum frost resistance value of 904 cycles was reached in 7.5% DWTS samples, whereas the control sample withstood 532 cycles.
5. Mechanical test results revealed a negative effect of the DWTS additive. The control sample had a compressive strength of 41.78 MPa. The strength of the 2.5% DWTS sample dropped to 36.39 MPa. A trend of decreasing compressive strength was observed with a higher DWTS content. The 7.5% DWTS had a compressive strength of 28.95 MPa. DWTS added at 12.5% by weight of cement reduced the compressive strength of modified mortars by more than 40%.

These studies suggest directions for further research on the replacement of the binder with raw DWTS. One of the directions is deeper research into the durability of DWTS-modified cementitious systems. Another research direction is to study the effect of DWTS on the physical, mechanical, and durability properties of modified mortars after thermal treatment of the additive at selected temperatures.

## Figures and Tables

**Table 1:** Composition of cement mortars, 1 m^3^.

Composition	Sand 0/4 fr., kg	Cement, %	Cement, kg	DWTS, g	DWTS, %	Water, g
DW-0	1500	100	500	0	0	275
DW-2.5	1500	97.5	487.5	12.5	2.5	275
DW-5	1500	95	475	25	5	275
DW-7.5	1500	92.5	462.5	37.5	7.5	275
DW-10	1500	90	450	50	10	275
DW-12.5	1500	88.5	437.5	62.5	12.5	275

## Data Availability

The original contributions presented in this study are included in the article. Further inquiries can be directed to the corresponding author.
